# PRP in OA knee – update, current confusions and future options

**DOI:** 10.1051/sicotj/2017004

**Published:** 2017-03-22

**Authors:** Mandeep S. Dhillon, Sandeep Patel, Rakesh John

**Affiliations:** 1 Department of Orthopaedics, Post Graduate Institute of Medical Education and Research Chandigarh 160012 India

**Keywords:** Early OA knee, Platelet-rich plasma, Hyaluronic acid

## Abstract

Positive results have been uniformly observed by various researchers for platelet-rich plasma (PRP) in early osteoarthritis (OA) knee in the past few years. PRP has clearly demonstrated its supremacy in comparison to hyaluronic acid (HA) and placebo in various clinical trials and is undoubtedly the best option available for symptomatic treatment in early OA. The release of growth factors from PRP occurs immediately and lasts for around three weeks and the clinical effect tends to wane down by the end of the year. Prolonged and sustained release of growth factors from platelets could possibly help in much better biological healing and sustained clinical effects. PRP in combination with biocompatible carriers could be one way of achieving this. Gelatin hydrogel PRP and chitosan PRP seem to be promising based on early in vitro studies and animal studies. PRP in combination with hyaluronic acid also seems to be additive. This article intends to discuss the present status of the PRP, confusions surrounding its use, upcoming trends and ideas for improvising PRP for use early OA knees based on available evidence.

## Introduction

Osteoarthritis (OA) of the knee is one of the commonest problems faced by ageing adults and in order to alleviate the pain and morbidity associated with OA, a variety of non-surgical treatment modalities ranging from oral chondroprotectives, intra-articular steroids to viscosupplements have been tried by pain physicians and orthopaedicians worldwide. Platelet-rich plasma (PRP) is evolving into a promising solution for various orthopaedic conditions like tendinopathies, non-union and arthritis of knee. The success of PRP in treating sports injuries in several high-profile sportsmen has contributed to the hype surrounding the PRP therapy, leading to increasing use of PRP for treating OA knees over the last seven years.

### Mechanism by which PRP works for knee OA

Osteoarthritis alters the normal joint metabolism favouring increased catabolism and decreased anabolism. Platelet alpha-granules contain and release numerous growth factors, including hepatocyte growth factor (HGF), vascular endothelial growth factor (VEGF), platelet-derived growth factor (PDGF) and transforming growth factor-b (TGF-b) [1], which could alter the changing joint mileu in OA.

PRP acts at various levels to alter the joint homeostasis.

In cartilage it decreases catabolism, improves anabolism and promotes chondral remodelling. Higher amounts of collagen II and prostaglandin (PG) synthesis have been documented by Akeda et al. [2] and Pereira et al. [3]. Increasing chondrocyte proliferation and production of matrix molecules have also been documented [4–7].

Synoviocytes are influenced by increased hyaluronic acid (HA) secretion [8], creating a more favourable and balanced state of angiogenesis [2, 9], and a decreased interleukin-1 (IL-1)-mediated rise in some matrix metalloproteinases (MMPs) [8, 9].

The apoptotic pathway of osteoarthritic chondrocytes is influenced as insulin-like growth factor 1 (IGF-1) in PRP may downregulate the expression of programmed cell death 5 (PDCD5) [10]. Lower levels of apoptosis were detected in in vivo studies by Mifune et al. [11] and the authors suggested that complex interaction of PRP within joint might positively influence chondrocyte apoptosis.

An overall downmodulation of the joint inflammation can explain the well-documented pain reduction, which is the most prominent and disabling symptom of knee OA. This could be through the regulation of nuclear factor kappa B (NF-κB) and cyclooxygenase-2 (COX-2), the principal actors of inflammatory cascade [3, 12, 13]. Other factors could be the inhibition of NF-κB transactivation activity mediated by HGF, a key cytokine present in PRP alpha-granules or an anti-inflammatory action by inhibiting monocyte-like cell chemotaxis [13]. Wu et al. [14]. showed that PRP counteracted the inflammatory cascade elicited by IL-1ß and tumor necrosis factor-alpha (TNF-α), showing an inhibition of IL-1ß, COX-2 and MMP-2 gene expression.

Lee et al. [15] noticed increase in mRNA levels of cannabinoid receptors CB1 and CB2 (receptors involved in analgesic and anti-inflammatory effects) and this could explain the analgesic effect of PRP.

### Preparation of platelet-rich plasma

PRP is the plasma fraction of autologous blood with platelet concentration above baseline. Platelet counts of 4–5 times of the baseline (1.5–4.5 × 10^5^/μL) label the product as PRP. Autogenous platelet gel, platelet enriched plasma (PeRP) and platelet-rich concentrate (PRC) are the synonyms for PRP.

There are various methods of PRP preparation and at least 25–30 ready-to-use kits are commercially available. Initial studies used PRP prepared in the laboratory by different techniques and based on these studies, the commercial kits have evolved. Broadly PRP can be prepared in two ways: “single-spinning” and “double-spinning”. Anitua et al. [16] had prepared PRP in a single-spin technique and open procedure which included micro-pipetting and named the product as EndoRet (plasma rich in growth factors). Patel et al. [17] also prepared PRP by the open technique which involved a single-spin, micro-pipetting and additional white blood cell (WBC) filtration, and their product was leucocyte-poor PRP. Kon et al. [18] have prepared PRP by the double-spin technique and cryopreserved the product and used it at three-weekly intervals.

Centrifugal forces and time, as well as the number of spins (double vs single) alter the PRP product in terms of platelet count and leucocyte concentration. Based on the variability in yield, it became necessary to classify PRP in order to compare studies and two classification systems have evolved. One is the Sports Medicine Platelet-Rich Plasma classification system by Mishra et al. [19], which takes into consideration the activation method (activated or not activated) and the leucocyte count (increased or absent) to divide the PRP into four types, with each having two further subtypes A and B based on the platelet concentration. The other international classification system is the PAW classification by DeLong et al. [20], which also takes into consideration the absolute platelet count (P1- low to P4- high), the manner of platelet activation and presence or absence of leucocytes.

### Platelet activation

Platelet activation can be achieved by different activators. Bovine or autologous thrombin is a traditional activator of platelets but there are concerns regarding its tolerability and adverse effects. Calcium chloride is the most common activator used in the majority of clinical studies. Collagen type-1 and batroxobin are some other activators which can be used. Rodeo et al. [21] showed that activated platelets release 70% of their growth factors (GFs) within the first 10 min and release most of the GFs within the next one hour. These growth factors are absorbed by the fibrin gel formed, which subsequently releases the various growth factors in a controlled way. The content of fibrin in the gel is the most important factor controlling subsequent release. Platelet concentration, fibrinogen concentration and the enzymes involved in procoagulant pathway influence the final fibrin content. The above factors regulate the duration of GF release at the injection site.

Studies aimed at improving the controlled delivery of GFs from PRP at the target site may bring out better results. Some novel approaches under consideration are the use of chitosan (scaffolds) [22, 23] and gelatin hydrogel as carriers of PRP. In a rabbit OA model, Saito et al. [24] demonstrated that gelatin hydrogel microspheres impregnated with PRP injections markedly suppressed OA progression both morphologically and histologically than the use of PRP alone.

### Should leucocytes be always filtered out?

The type of PRP to be used is another topic of debate. The confusion is between leucocyte-rich and leucocyte-poor PRP. We raised our concern regarding this previously [25]. The initial hypothesis that leucocytes could be proinflammatory inside the joint (due to supposed deleterious effects of proteases and reactive oxygen species released from white cells) has been subsequently corroborated.

1. Pifer et al. [26] showed in an in vivo study that PRP with leucocytes contains MMP-2, -3 and -9, which is released over a period of at least six days, and can be deleterious.
2. Braun et al. [27] compared the effects of leucocyte-rich PRP (LR-PRP), leucocyte-poor PRP (LP-PRP), red blood cell (RBC) concentrate and platelet-poor plasma (PPP) and concluded that “Treatment of synovial cells with LR-PRP and RBCs resulted in significant cell death and proinflammatory mediator production”.
3. Dragoo et al. [28], in a rabbit study, showed that the (LR-PRP) group had more undesirable side effects owing to greater inflammatory reactions following injection at the lesion site than at the LP-PRP group.
4. Filardo et al. [29] – These authors were the only ones to conduct a clinical trial comparing two different PRP preparations: high-concentrate leucocyte-rich PRP versus low concentrate leucocyte-free PRP. They treated 144 patients and evaluated up to 12 months and comparable positive results were obtained in both treatments, with the only difference being that the PRP leucocyte group suffered from more swelling and pain reaction immediately after the injections.

To add to the confusion, there are some in vivo studies which document some beneficiary effects of leucocyte released products for the OA knee. Cavallo et al. [30] in their in vitro study noticed that chondrocyte proliferation and hyaluronan secretion were more prevalent in L-PRP than in P-PRP. Riboh et al. [31] in a recent meta-analysis compared clinical outcomes and rates of adverse reactions between LP-PRP and LR-PRP for the OA knee. They concluded that both are clinically effective over HA and placebo. LP-PRP and LR-PRP had similar safety profiles, and adverse reactions to PRP may not be directly related to leucocyte concentration.

Thus, there is still a need of research on this topic so as to standardize the concentration of leucocytes needed in ideal PRP preparation injected in OA.

### What specific type of PRP is ideal for Knee OA?

Based on the available literature, there are some answers and but more questions which need to be answered.

Different PRP preparations – Magalon et al. [32] studied five different commercial PRP preparations in a single donor model and noticed significant biological variation in the PRP product among different preparations and postulated this to be a reason for the variability of results in PRP studies.

Intra-individual variations were observed by Mazzocca et al. [33] in the same individual, and there were variations in the PRP yield by the same method in samples drawn at different time periods.

In the debate about fresh PRP versus freeze thawed PRP, the fresh PRP appears to be better. Storing platelets in freezing conditions can alter the morphology and decrease the functional properties of platelets by degranulation of alpha-granules [34]. We had expressed our concerns regarding cryopreservation of PRP in our initial work [25]. However, freeze thawing PRP is better in terms of patient compliance as the PRP can be prepared in a single sitting. Roffi et al. [35] studied the effect of freezing/thawing on the PRP molecule release, and its effects on the metabolism of chondrocytes and synoviocytes. They noticed decreased protein level secretion in the freeze thawed PRP but the gene expression in cultured chondroctyes and synoviocytes was similar to that in fresh PRP. They concluded that PRP cryopreservation is a safe procedure, which sufficiently preserves PRP quality and its ability to induce proliferation and the production of Extra Cellular Matrix (ECM) components in chondrocytes and synoviocytes.

For knee OA, leucocyte-poor PRP appears to be better than leucocyte-rich PRP.

### Clinical studies

Over 35 clinical trials have been conducted in the past seven years, which reflects the growing interest in exploring PRP as treatment modality in the OA knee. It is surprising to notice that in all previous studies (case series as well as comparative studies), superiority of PRP has been demonstrated in alleviating pain symptoms and improving knee scores.

Sanchez et al. established the safety of autologous PRP for intra-articular use in the first PRP trial in 2008 [36]. It was followed by subsequent studies which compared PRP with hyaluronic acid (HA) and demonstrated the safety profile and beneficial effects of PRP in the OA knee. Spakova et al. [37] compared three PRP injections with three hyaluronic acid injections in their randomized control trial (RCT) on 120 patients and concluded the effectiveness and safety of autologous PRP in early osteoarthritis knee (Kellgren and Lawrence Grades 1, 2 or 3 Osteoarthritis). Better Western Ontario and McMaster Universities Arthritis Index (WOMAC) scores and Numerical Rating Scale (NRS) were noted in the autologous PRP group in comparison to the HA group. Cerza et al. [38] in their RCT on 120 patients compared four PRP injections at one-week interval with low molecular weight hyaluronan (LMW-HA) and observed better improvement of WOMAC scores at 24 weeks in the PRP group. They did not find any correlation with the grade of OA.

Kon et al. [18] treated 91 patients with three PRP injections at three-week intervals (freeze thawed PRP) and noted improvement at six and 12 months from baseline in International Knee Documentation Committee (IKDC) and visual analogue scale (VAS) scores, with a tendency of worsening between six and 12 months. In a subsequent comparative study, Kon et al. [39] observed better symptom control and sustained effects (better IKDC and EQ-VAS scores) in autologous PRP group (three injections at two-week intervals) compared to high molecular weight hyaluronan (HMW-HA) injections (50 patients) and LMW-HA injections (50 patients). They have established good outcomes (IKDC scores) of intra-articular PRP in early degenerative cartilage lesions. They have quoted on better results in younger patients, low body mass index (BMI) patients and those with less degree of cartilage degeneration. They also followed the same patients for two years and noticed sustained improvement compared to baseline in the PRP group than HA, with a slight worsening after the first year [40]. However in their recent RCT, they found a similar benefit in both HA and PRP groups in early OA [41].

Sanchez et al. [42] in their RCT of 176 patients with Ahlbacks grade 1–3 OA compared three PRP injections at one-week intervals (79 patients) with those of HMW-HA (74 patients). The primary outcome measure was the percentage of patients having a 50% decrease in the WOMAC pain subscore. The secondary outcome measures being other WOMAC subscores, Lequesne index and Osteoarthritis Research Society International (OARSI) responders. They noticed better outcomes in the PRP group at 24 weeks in respect to primary outcome. No differences for secondary outcome measures and amount of acetaminophen consumption were observed.

Similarly better outcomes were documented in the PRP group in comparison to HA groups at six months by Li et al. [43] and Say et al. [44] in their prospective studies.

Patel et al. were the first to compare normal saline (physiological control) with PRP and established the superiority of PRP over placebo as manifested by improved WOMAC scores which were sustained at six months [17]. They noticed that patients were experiencing benefits as early as 18 days and also noted a slight worsening of benefits by six months, based on which they hypothesized that anti-inflammatory role could be the reason for the clinical effect, as for chondral remodelling it would have required much more time and would have given much sustained results [45].

There is also a lot of confusion regarding the dosage schedule of PRP for OA knees. Initial studies used three injections at three weekly interval (without any rational though); probably in a bid to compare with HA which is used similarly. The literature is confusing, with studies available which have used two injections, three injections to four injections. The duration between injections is also variable (one week to four weeks). We were the first to compare two different PRP injection groups, and found that single injection was as good as two injections of PRP, shown by similar improvement in WOMAC scores [17]. Recently Görmeli et al. [46] in their double-blind placebo-controlled randomized trial noted a statistically significant improvement in the IKDC and EQ-VAS scores in all the treatment groups compared with the control group (Normal Saline). The knee scores of patients treated with three PRP injections were significantly better than those patients of single PRP and HA groups.

Another alternative is to use PRP at yearly intervals or when the patient demands it again after the effect wanes out. Gobbi et al. [47] have used PRP at yearly interval and established the clinical efficacy. A lot more research in this direction needs to be carried out as to how long we can prolong the pain-free status with multiple yearly injections.

Hart et al. [48] have used another interesting approach in their trial wherein they compared PRP (50 patients) with 1% mesocaine (50 patients) in knee articular damage grade 2 (fibrillation) and grade 3 (fissuring and fragmentation). The PRP group received a total of nine injections within a year. The first six injections (loading dose) at weekly interval followed by a three-month gap; followed by three injections at three month interval (maintenance dose). They noticed a better improvement of PRP groups at 12 months with respect to IKDC, Tegner, Lysholm and Cincinnati scores. However, no significant influence on cartilage was observed in magnetic resonance imaging (MRI). So, no clear benefit of such PRP loaded procedure could be validated.

Hassan et al. [49] looked at 20 patients with mild to moderate OA, giving 5 mL PRP at monthly intervals for six months (six injections); they noticed significant improvement in knee stiffness, IKDC scores and VAS scores compared to baseline. Maximal improvement was obtained in patients with young age, less BMI and short disease duration.

Majority of the previous studies have included early OA for PRP therapy and consistently showed benefits in terms of symptomatic improvement. Kon et al. [18, 39] and Hassan et al. [49] have compared early OA with late OA and found better results in early OA. Recently Sánchez et al. [50] and his team have described a novel approach of PRP delivery in severe OA by intraosseous infiltration of PRP in subchondral area of femoral condyle, tibial condyle and patella. They also simultaneously gave intra-articular injections of PRP for addressing synovial and cartilage pathology in OA.

Another interesting approach towards PRP administration in OA is the use of photo-activated PRP (PA-PRP). Paterson et al. [51] in a randomized controlled pilot study (23 patients) observed the safety profile and feasibility of use of PA-PRP in OA knee. Better scores were observed in comparison with the HA group. However, studies are required to compare the PA-PRP with PRP to show any additional effect of photo-activation over conventional PRP.

There have been a few studies [52–54] demonstrating the PRP efficacy over HA in hip OA. Mei-Dan et al. [55] demonstrated better outcomes in the PRP group at 28 weeks in talar osteochondral lesions.

With the availability of commercial PRP kits in market, more and more people can receive the treatment. However, it is advisable for the clinicians to not get carried away with the initial results and to keep track of the patient’s outcome so as to contribute to the existing literature. It is also advisable to look at the yield and the product obtained to classify the PRP type.

Anitua et al. [56] had postulated that PRP in combination with HA may be synergistic, by enhancing the migratory potential of fibroblast based on her in vitro studies. The same has also been supported by Marmotti et al. in his in vitro study [57]. Both HA and PRP are biological approaches and their use may be critical in the initial phase of OA environment where tissue healing may benefit. Based on these concepts Andia et al. [58] have expressed that HA+PRP may be better than PRP alone. Dallari et al. [54] in their RCT in hip primary OA compared ultrasound guided injections of PRP, HA and HA+PRP and noticed a significant improvement in WOMAC and Harris Hip score in the PRP group over HA. However, the addition of PRP+HA did not lead to significant improvement in pain symptoms. A recent RCT by Lana et al. [59] compared HA+PRP versus PRP versus HA in mild to moderate knee OA and noticed better outcomes in the HA+PRP combination over HA alone up to one year and over PRP alone up to three months. They also noted better functional outcomes in the first 30 days after treatment in the combination group over HA and PRP alone groups. Clinical studies on combination therapy are limited and further well-designed studies with a larger sample size are required before a definitive comment can made. Several key aspects concerning molecular weight, ideal combination and dosage schedule of both need to be evaluated before conducting clinical trials. HA+PRP definitely seems like a good future option.

## Conclusion

The present state of knowledge holds promise for PRP of certain specifications for pain management in the early OA knee. PRP has consistently been shown by various clinical studies to be superior to HA. Nevertheless, a lot of grey areas remain in our understanding of PRP and OA, and many more focused clinical and in vitro studies are required. HA+PRP seems to be an evolving future trend. Researchers are also focused on developing a better PRP product by combining it with various molecules such as gelatin, chitosan and others. PRP is definitely there to stay for OA therapy use in future.

## Conflict of interest

The authors declare no conflict of interest in relation with this paper.
